# Characteristic of bioclimatic conditions in Poland based on Physiologically Equivalent Temperature

**DOI:** 10.1007/s00484-023-02557-6

**Published:** 2023-10-07

**Authors:** Arkadiusz M. Tomczyk, Andreas Matzarakis

**Affiliations:** 1 Department of Meteorology and Climatology, Institute of Physical Geography and Environmental Planning, Adam Mickiewicz University, B. Krygowskiego 10, 61–680 Poznań, Poland; 2 Research Centre Human Biometeorology, Deutscher Wetterdienst, Freiburg, Germany; 3 Institute of Earth and Environmental Sciences, Faculty of Environment and Natural Resources, Albert-Ludwigs-University, Freiburg, Germany

**Keywords:** Bioclimatic conditions, PET, Climate change, Poland

## Abstract

The aim of the study was to characterise bioclimatic conditions in Poland in the times of progressing warming. This type of research permits the verification whether the progressing climate warming also translates into a change in bioclimatic conditions. This study was based on data obtained for the period 1966–2021 from 37 synoptic stations located in Poland. All the data series were uniform and obtained from the data base of the Institute of Meteorology and Water Management—National Research Institute (IMGW-PIB). The study revealed high variability of bioclimatic conditions in Poland both in spatial and in temporal terms. The lowest mean annual PET values were recorded in the north and north-east of the country and the highest in the south-west of Poland. The study revealed changes in the frequency of occurrence of days with cold and heat stress, as well as days with no thermal stress. The most intensive changes were determined for days with cold stress. A decrease in the number of days in this category translated into an increase in the number of days with no thermal stress and days with heat stress.

## Introduction

The currently progressing warming (IPCC 2021) translates into a transformation of bioclimatic conditions. Research on such conditions is recommended to employ indices that consider various meteorological parameters, because the human organism in the natural environment is affected not only by air temperature but also by air humidity, wind speed, etc. The literature distinguishes between simple and complex indices. The former group of indices includes those that describe the effect of several individual meteorological parameters on the human organism, namely air temperature, wind speed, and air humidity. They do not directly refer to the physiological response of the organism, and their impact on the human organism is considered based on the analysis of thermal perception or heat stress (Błażejczyk 2004; Błażejczyk et al. 2012). This group of indices includes among others the Humidex (Masterson and Richardson 1979), Effective Temperature (ET) (Houghton and Yaglou 1923), and Apparent Temperature (AT) (Steadman 1984). The second group includes indices based on different models of human heat balance. According to Błażejczyk et al. (2012), the characteristics of the thermal environment from the point of view of thermal-physiological conditions require the application of the complete model of heat budget considering all mechanisms of heat exchange. Unlike in the case of such indices, simple indices can never fulfil the essential requirement that for each index value there must always be a corresponding meaningful thermo-physiological state (strain intensity), regardless of the combination of the input meteorological values (Błażejczyk et al. 2012). Due to this, their applicability is limited, and the results are frequently not comparable. The second group of indices include among others: Physiological Strain (PhS) and Physiological Subjective Temperature (PST) (Błażejczyk 1994, 2005, 2007; Błażejczyk and Matzarakis 2007; Błażejczyk et al. 2012), although the Universal Thermal Climate Index (UTCI) (Błażejczyk et al. 2010) and Physiologically Equivalent Temperature (PET) (Mayer and Höppe 1987; Höppe 1999; Matzarakis et al. 1999) have been recently applied most frequently.

Currently available studies in the scope of biometeorology and bioclimatology in Poland have been primarily conducted based on UTCI. Such studies include those covering the entire country (Tomczyk and Owczarek 2020; Wereski et al. 2020; Krzyżewska et al. 2021a, b; Kuchcik et al. 2021; Owczarek and Tomczyk 2022; Tomczyk and Bednorz 2023; Tomczyk et al. 2023), as well as selected regions or cities (Kolendowicz et al. 2018; Miszuk 2021; Rozbicka and Rozbicki 2021). Among numerous publications, the paper by Błażejczyk and Twardosz (2023) is particularly interesting. The authors analysed the occurrence of heat and cold stress in Kraków in the period 1826–2021. Bioclimatic research based on such long data series can be considered unique not only at the national but also at the global scale. In recent years, researchers have been particularly focusing on the occurrence of extreme situations, i.e. those causing at least strong heat stress (Owczarek 2019; Tomczyk and Owczarek 2020; Krzyżewska et al. 2021a, b; Miszuk 2021; Tomczyk 2021; Tomczyk et al. 2023) or strong cold stress (Wereski et al. 2020; Miszuk 2021; Owczarek 2021; Owczarek and Tomczyk 2022; Tomczyk et al. 2023), or the assessment of biometeorological conditions during heat waves (Krzyżewska et al. 2019; Tomczyk 2019).

The scarce studies from Poland have been so far employing PET. Among the first such publications, the paper regarding the assessment of the bioclimatic diversity of Poland by Błażejczyk and Matzarakis (2007) deserves particular attention. In recent years, the index has been used for the assessment of biometeorological conditions during heat waves in Poland (Tomczyk et al. 2020). The index has been most frequently applied in the assessment of biometeorological conditions in Germany (Matzarakis and Endler 2010; Matzarakis 2013) and in other regions of Europe, e.g. in Ukraine (Shevchenko 2021; Shevchenko et al. 2022), Greece (Nastos and Matzarakis 2012), Austria (Ferrari et al. 2015), and Serbia (Basarin et al. 2018; Pecelj et al. 2021; Milošević et al. 2023). Moreover, research based on PET has been conducted in Nigeria (Omonijo 2017), Japan (Matzarakis et al. 2019), and India (Bal and Matzarakis 2022).

As mentioned above, bioclimatic conditions in Poland have been repeatedly analysed. There is, however, still no coherent and complex assessment of bioclimatic conditions based on a long data series from the entire country that would also cover the recent years, characterised by intensively progressing climate warming. It is therefore justified to implement this type of research. According to Rozbicka and Rozbicki (2021), both daily/inter-day and long-term bioclimatic conditions have a considerable effect on the quality of life in the urban environment.

The paper objective was to characterise bioclimatic conditions in Poland in the times of progressing warming. The assumed objective was implemented through the determination of bioclimatic conditions in the long-term, annual, and seasonal scale, as well as through the determination of the direction and rate of their changes in the analysed period 1966–2020. This type of research permits the verification whether the progressing climate warming also translates into a change in bioclimatic conditions.

## Data and methods

**Fig. 1:**
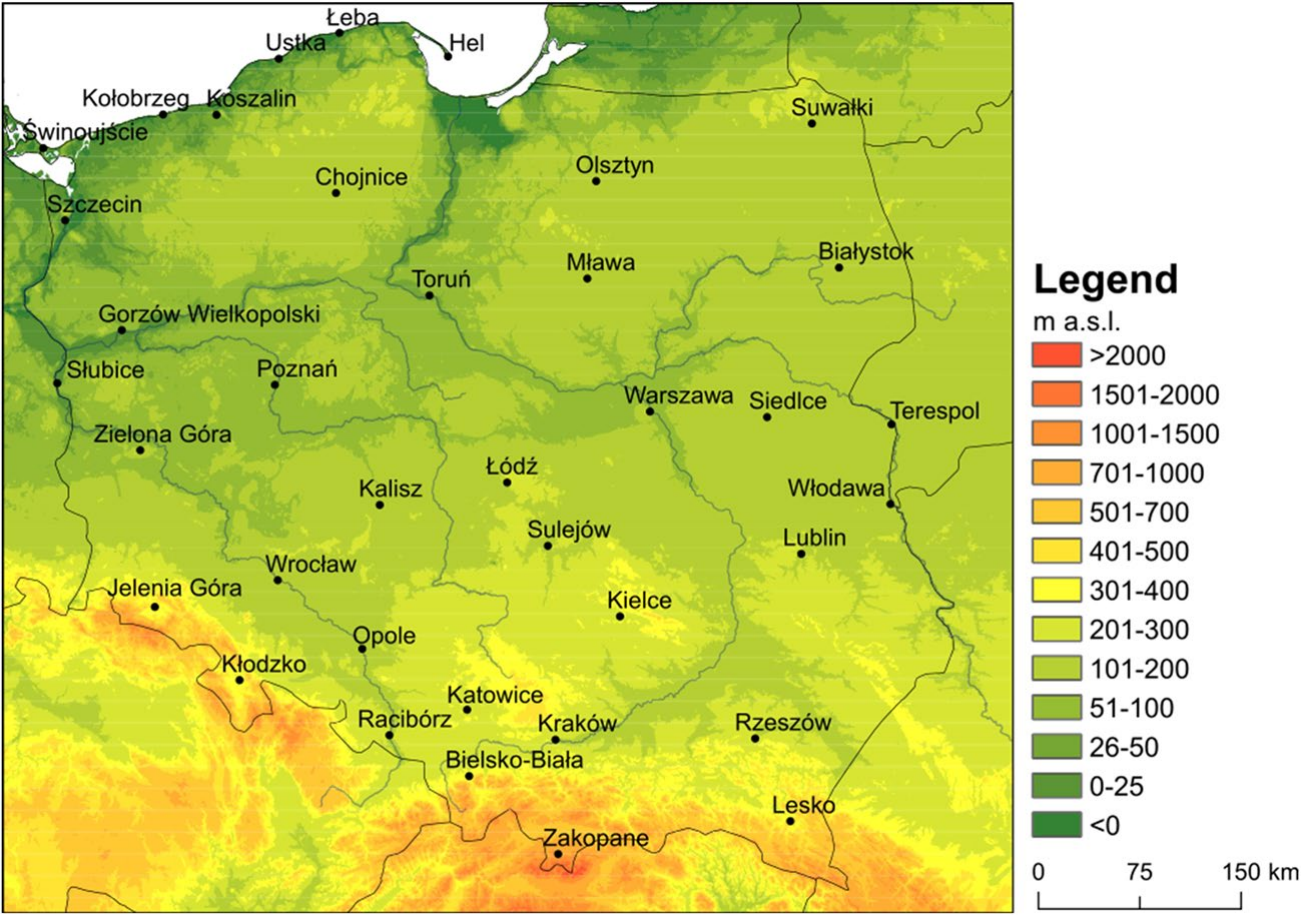
Location of the synoptic stations in Poland

The basis for the study was data from 37 synoptic stations located in Poland from the period of 1966–2021 (from 2021 for two winter months: January and February) (Fig. 1). The stations are located outside the city centres. It employed data series from 12:00 UTC such as air temperature (°C), relative humidity (%), wind speed (m∙s^−1^), and total cloudiness (okta). This hour is a standard observation term providing data for research in the scope of biometeorology and bioclimatology. This time of day corresponds to most types of outdoor human activity in Poland. All the data series were uniform and obtained from the data base of the Institute of Meteorology and Water Management—National Research Institute (IMGW-PIB). Wind speed was estimated at 1.1 m, which is the gravity centre of the human body. The reduction of the wind speed has been performed according to previous human-biometeorological studies (Matzarakis et al. 2009).

The data provided the basis for the calculation of the human thermal index PET. PET is defined as the air temperature at which, in a typical indoor setting (without wind or solar radiation), the energy balance of the human body is balanced with the same core and skin temperature as under the complex outdoor conditions to be assessed (Mayer and Höppe 1987; Höppe 1999; Matzarakis et al. 1999; Matzarakis and Amelung 2008). PET is expressed in degree Celsius (°C), facilitating its interpretation. PET is one of the most used index for the quantification of thermal comfort and heat stress (Potchter et al. 2018, 2022). The settings for thermo-physiological are standard for comparison and harmonisations. The calculation was conducted by means of the RayMan model (Matzarakis et al. 2007, 2010, 2021; Matzarakis and Endler 2010; Matzarakis and Fröhlich 2018; Fröhlich et al. 2019). It is a micro-scale model developed to calculate radiation fluxes in simple and complex environments. This allows the calculation of Tmrt, which is an important input parameter in the calculation of thermal biometeorological indices.

The calculated PET values provided the basis for calculating the mean annual and seasonal (spring: March–May; summer: June–August; autumn: September–November; winter: December–February) index value. Mean PET values were also calculated for shorter subperiods. Because the analysis covered 55 years, the period was divided into five decades in the years 1971–2020 and a 5-year period (1966–1970), although it was not considered due to the incomplete number of years. Only full decades were considered in the analyses. Next, changes in the calculated values in the multiannual period were investigated. The rate and direction of changes were assessed by means of linear regression, and the statistical significance of trends was verified by means of a *t*-Student test.

In the following stage, based on the calculated values, the PET category was determined (Table 1) for each day, followed by the determination of the frequency of occurrence of these categories in the study period in particular stations. Moreover, changes in the annual number of days with cold stress, days with no thermal stress, and days with heat stress in the years 1966–2020 were analysed, and the statistical significance was determined by means of a non-parametric Mann–Kendall test (Mann 1945) at a significance level of 0.05.

**Table 1:** PET for different levels of thermal perception and physiological stress on human beings (during standard conditions where the heat transfer resistance of clothing is 0.9 clo and the internal heat production is 80 W) (Matzarakis et al. 1999)

PET (°C)	Thermal perception	Grade of physical stress
< 4.0	Very cold	Extreme cold stress
4.1–8.0	Cold	Strong cold stress
8.1–13.0	Cool	Moderate cold stress
13.1–18.0	Slightly cool	Slight cold stress
18.1–23.0	Comfortable	No thermal stress
23.1–29.0	Slightly warm	Slight heat stress
29.1–35.0	Warm	Moderate heat stress
35.1–41.0	Hot	Strong heat stress
> 41.0	Very hot	Extreme heat stress

The maps were made in the Surfer13 program. The kriging method was used for interpolation.

## Results

**Fig. 2:**
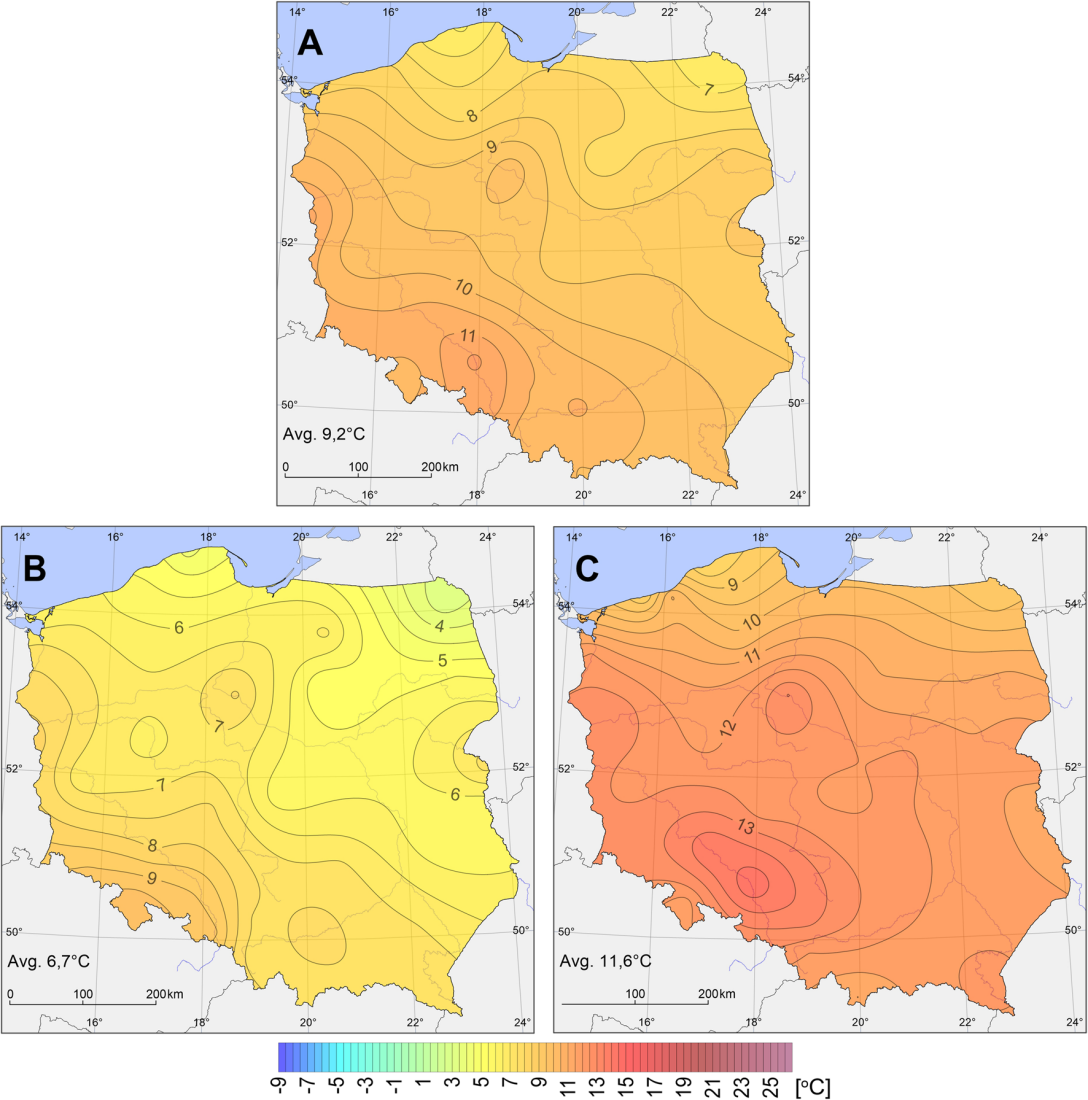
Mean annual PET in Poland for 12:00 UTC: in the years 1966–2020 (**A**), mean PET in 1980 (**B**) and 2018 (**C**)

In the years 1966–2020, mean annual PET value in Poland was 9.2 °C. The index value showed spatial variability and increased from the north and south-east (below 7 °C) towards the south-west (more than 10.0 °C, and in the upper course of the Oder River even more than 11.0 °C) (Fig. 2). In the study period, high PET fluctuations were recorded from year to year although the variability was comparable throughout the area, as indicated by the standard deviation values. In 91% of stations, the values were within a range of 1.2–1.5 °C, and the lowest value was observed in Ustka (0.9 °C).

**Fig. 3:**
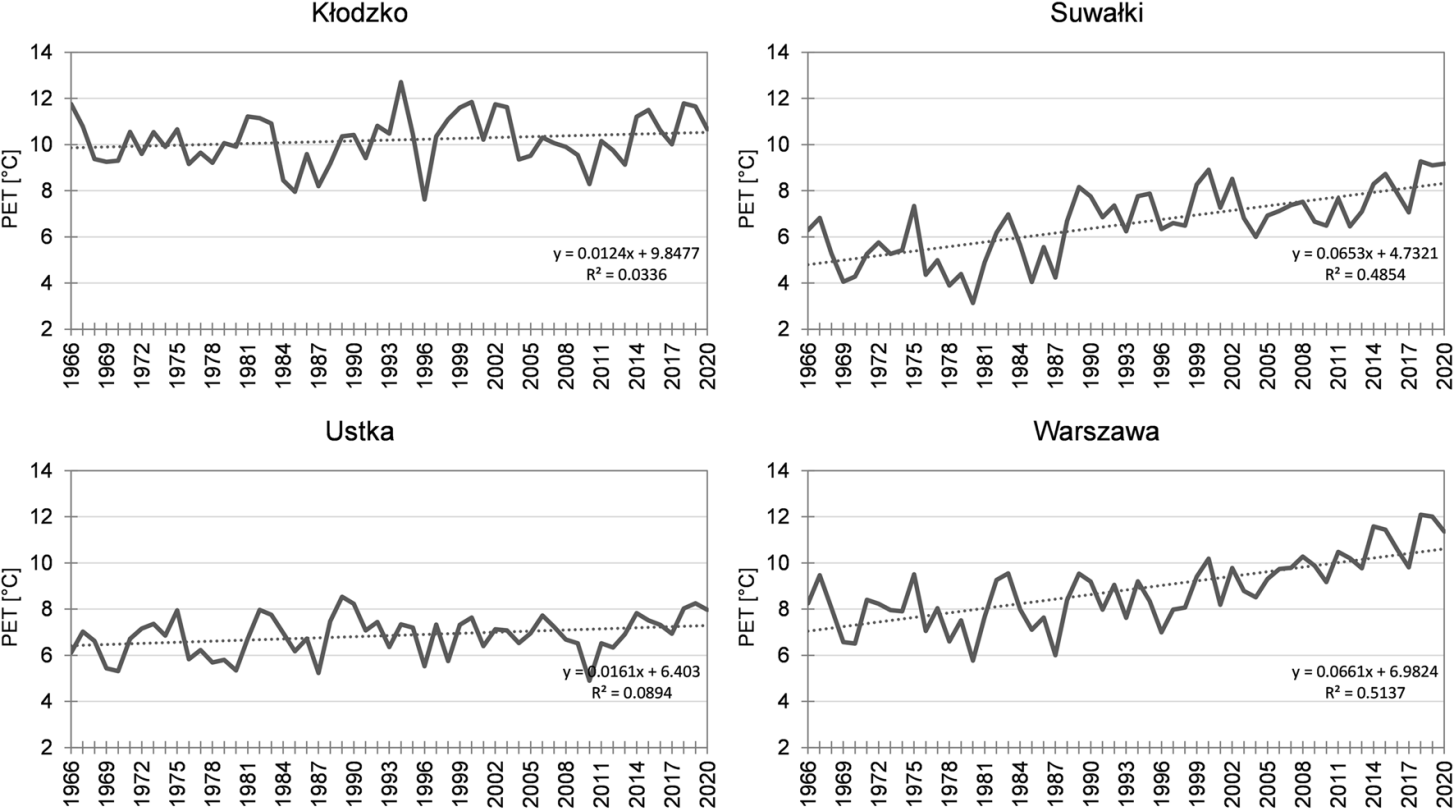
Mean annual PET in the years 1966–2020 (for 12:00 UTC) in Kłodzko, Suwałki, Ustka, and Warszawa

In most stations (51%), the lowest mean annual PET value was recorded in 1980, with an average for the entire area of only 6.7 °C. In that year, the index values evidently increased from the north-east towards the south-west of the study area (Fig. 2). In particular stations, mean annual PET value varied from 3.1 °C in Suwałki to 9.9 °C in Kłodzko (Fig. 3). An equally high value was recorded in Raciborz (9.1 °C). In those two stations, the lowest PET for the entire multiannual period was recorded not in 1980, but in 1996 (with values of 7.6 °C and 8.5 °C, respectively). The year with the second lowest PET value was 1987 (mean for the entire area 6.9 °C), when the minimum for the entire period was recorded in 30% of stations.

In 76% of stations, the highest mean annual PET value was recorded in the twenty-first century. Considering the highest index value calculated for the entire area, the record year throughout the multiannual period was 2018, with a mean of 11.6 °C. An increase in PET values occurred from the north towards the south-west of the country (along the Oder River valley) (Fig. 2). The range of fluctuations of the analysed index was from 8.0 °C in Ustka to 14.4 °C in Opole. In that year, the highest PET value throughout the multiannual period was recorded in 35% of stations, and in the following year in 30% of stations. In 2019, the mean for the entire area was 11.5 °C and varied from 8.2 °C in Łeba to 14.1 °C in Opole.

During the research period 1966–2020 (except for Raciborz), an increase in PET values was observed in the territory of Poland. The most intensive changes were recorded in the area stretching from the north-west to the north-east through the middle part of the country. An increase in PET values ranged from 0.12 °C/10 years in Kłodzko to 0.66 °C/10 years in Świnoujście and Warsaw (Fig. 3). Moreover, at least 0.60 °C/10 years was recorded in Suwałki, Mława, Katowice, Łódź, and Terespol. In 92% of the stations, the changes were statistically significant. The progressing warming was also evident in mean PET in particular decades (Table 2). In all stations, the highest mean index value was recorded during the last of the analysed decades (2011–2020). Moreover, in two stations, the same value was also observed in the years 1991–2000. In 84% of stations, the lowest mean value was determined for the first decade (1971–1980), and in one station, the same value was recorded in the subsequent decade.

**Table 2:** Mean annual PET in Poland in the years 1966–2020 (for decades)

Station	1966–1970	1971–1980	1981–1990	1991–2000	2001–2010	2011–2020	Station	1966–1970	1971–1980	1981–1990	1991–2000	2001–2010	2011–2020
Białystok	8.5	6.9	8.8	8.8	8.9	9.9	Opole	11.5	10.6	11.8	11.2	11.6	13.0
Bielsko-Biała	9.6	9.1	9.8	9.5	11.0	11.7	Poznań	8.4	8.2	9.0	9.7	9.7	10.8
Chojnice	7.0	7.1	7.7	7.7	7.9	8.8	Racibórz	12.8	11.5	10.6	10.8	10.7	11.8
Gorzów Wielkopolski	9.3	8.8	9.2	10.1	10.7	11.6	Rzeszów	8.8	8.8	9.6	10.1	10.1	11.1
Hel	6.8	7.0	7.6	7.8	8.1	8.8	Siedlce	8.5	8.1	8.1	8.5	9.2	10.5
Jelenia Góra	9.5	9.9	10.9	11.6	10.8	11.6	Słubice	10.2	10.5	10.7	11.8	11.6	11.9
Kalisz	10.1	9.2	9.1	9.2	9.5	10.7	Sulejów	8.5	8.2	8.7	9.1	9.6	10.8
Katowice	9.4	9.3	9.8	10.8	10.9	12.1	Suwałki	5.3	5.0	6.0	7.3	7.1	8.1
Kielce	9.7	8.6	8.9	9.5	9.8	11.0	Szczecin	8.1	8.7	9.3	9.7	9.6	10.9
Kłodzko	10.1	9.9	9.7	10.6	10.1	10.6	Świnoujście	7.1	6.9	7.2	8.5	9.2	9.7
Kołobrzeg	6.6	7.2	8.2	8.5	9.1	9.4	Terespol	7.6	8.0	9.3	9.2	9.7	10.8
Koszalin	6.6	7.2	7.3	8.7	8.7	9.3	Toruń	8.9	8.9	9.3	9.7	10.2	11.5
Kraków	9.5	9.7	10.5	11.0	10.9	11.5	Ustka	6.1	6.5	7.2	6.9	6.7	7.4
Lesko	9.2	9.2	8.6	9.1	9.9	11.1	Warszawa	7.8	7.7	8.2	8.5	9.3	10.9
Lublin	8.7	7.8	8.4	8.7	9.5	10.4	Włodawa	8.6	8.0	7.9	8.5	9.0	10.0
Łeba	6.4	5.7	5.6	6.4	6.9	7.5	Wrocław	9.8	10.2	10.5	10.7	10.9	12.5
Łódź	7.6	7.6	8.5	9.0	9.3	10.5	Zakopane	9.3	9.7	9.8	10.4	9.8	10.6
Mława	6.9	6.3	7.2	7.8	8.2	9.4	Zielona Góra	8.9	8.8	8.9	9.5	9.9	11.0
Olsztyn	7.3	7.3	8.4	8.6	8.5	9.5							

**Fig. 4:**
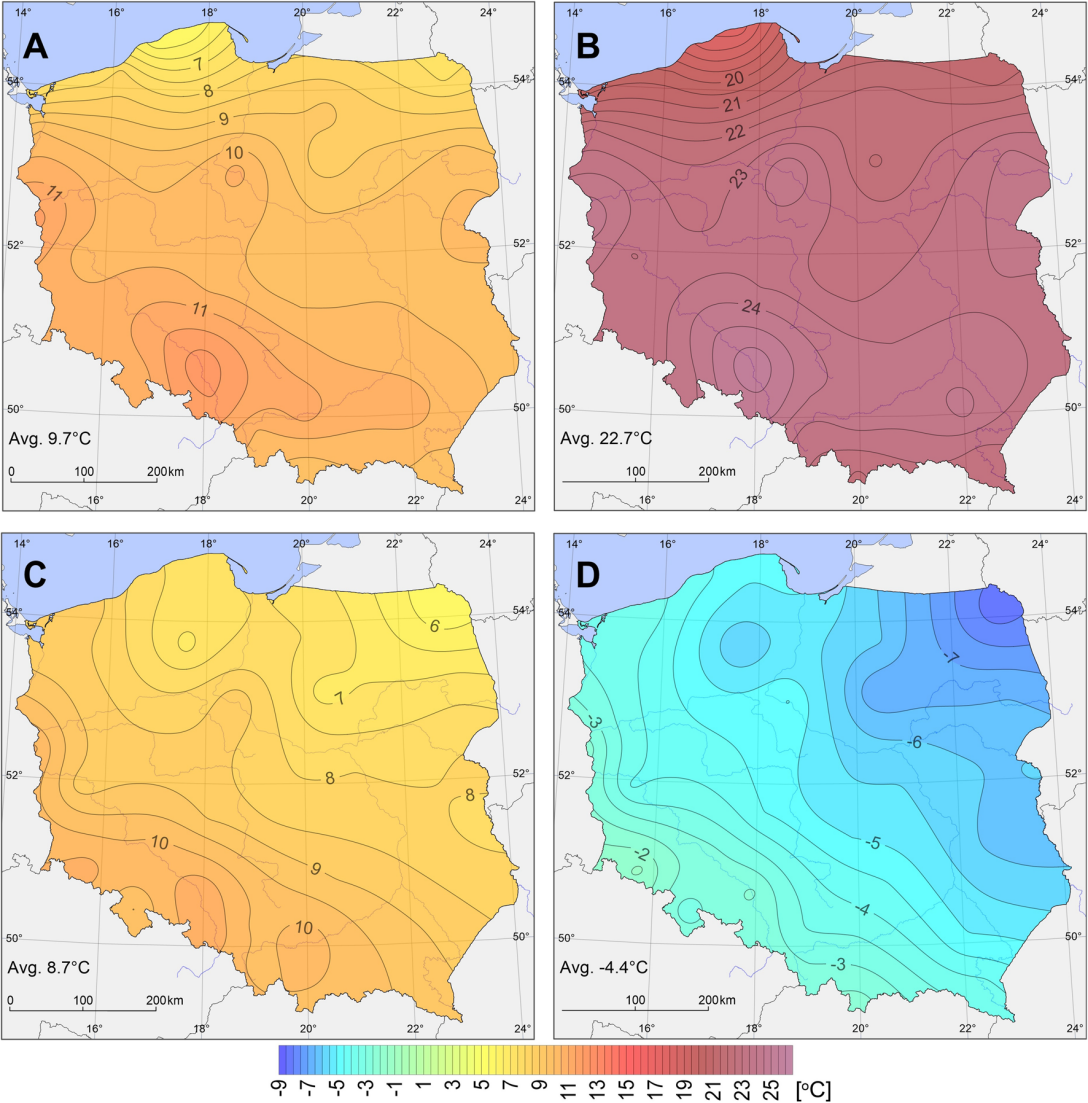
Mean PET in Poland: in spring (**A**), summer (**B**), autumn (**C**), and winter (**D**) in the years 1966–2020 (for 12:00 UTC)

**Fig. 5:**
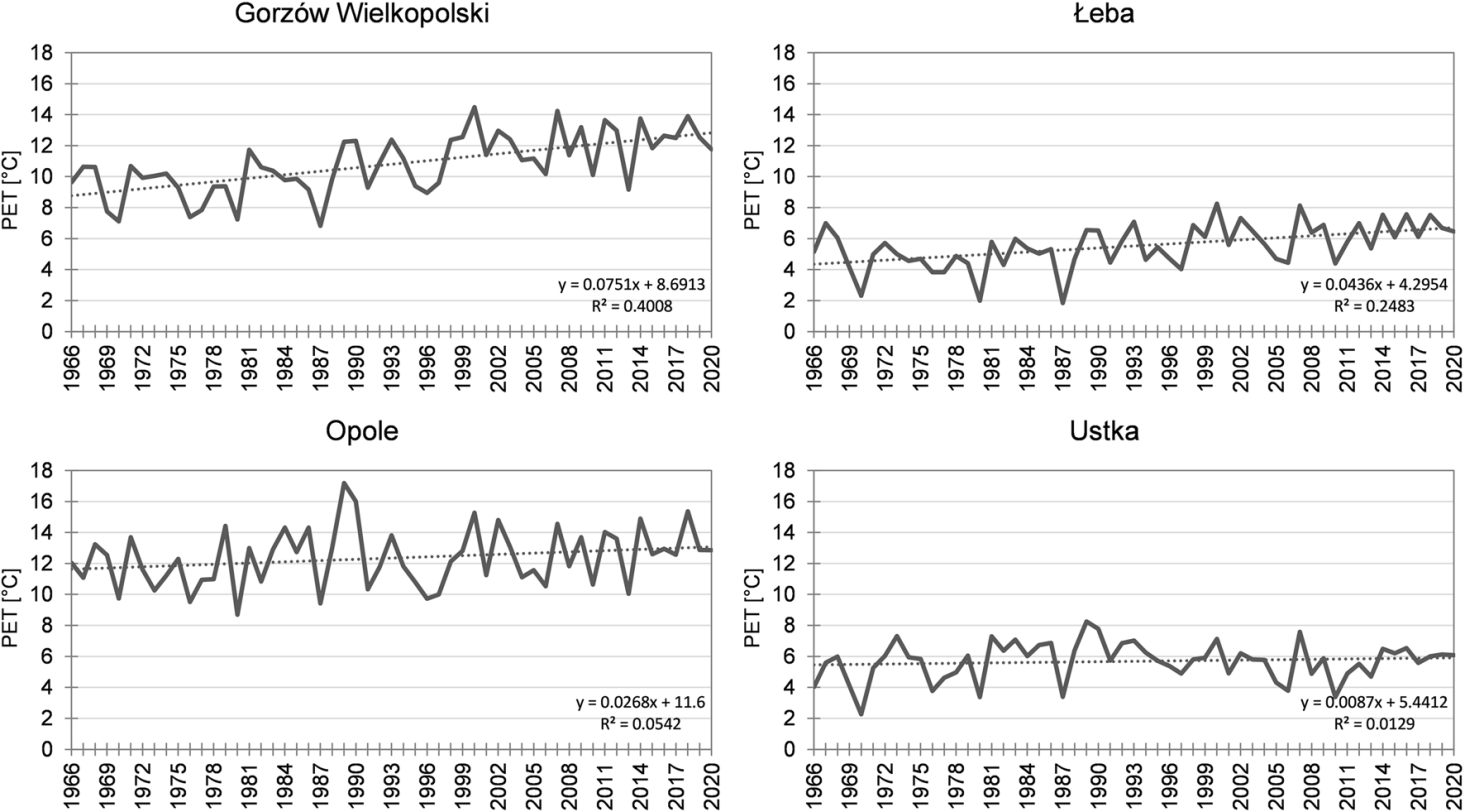
Mean PET in spring in the years 1966–2020 (for 12:00 UTC) in Gorzów Wielkopolski, Łeba, Opole, and Ustka

In spring in the period 1966–2020, mean PET value was 9.7 °C. The index value increased from the north towards the south-west of the country and varied from 5.5 °C in Łeba to 12.4 °C in Opole (Fig. 4). During the research period 1966–2020, the lowest mean PET value for the entire area observed in 1987 was 6 °C. In that year, minimum value throughout the multiannual period was recorded in the highest number of stations (43%). In particular stations, the index value fluctuated from only 1.8 °C in Łeba to 9.4 °C in Opole (Fig. 5). Equally low mean PET values were recorded in 1970 (minimum in 32% of stations) and 1980 (minimum in 21% of stations). The highest mean PET value was observed in 2000, reaching 12.7 °C for the entire area. In that year, the index value varied from 7.1 °C in Ustka to 15.3 °C in Opole and 15.2 °C in Słubice. Approximate bioclimatic conditions were observed in 2018, when the mean for the entire country was 12.5 °C and varied from 6.0 °C in Ustka to 15.4 °C in Opole (Fig. 5). Maximum PET value throughout the multiannual period for spring was recorded in 30% of stations in 2000 and in 27% of stations in 2018. In the analysed period, an increase in PET values was observed in spring (except for Raciborz). The most intensive changes (> 0.50 °C/10 years) were recorded in the belt stretching from the north-west towards the south-east through the central regions of the country, with the exception of the eastern part of the coast. An increase in values varied from 0.09 °C/10 years in Ustka to 0.82 °C/10 years in Świnoujście. Equally considerable changes occurred in Gorzów Wielkopolski (0.75 °C/10 years). In 84% of the stations, the changes were statistically significant. In 32% of stations, changes in PET recorded in spring were the greatest among all the seasons. Like in the case of mean annual values, the highest mean PET were also recorded in spring over the last decade (2010–2020), as observed in 86% of stations (Table 3). In 95% of stations, the lowest mean index value was determined in the years 1971–1980 (in one station, the same value was recorded in the one but last decade).

**Table 3:** Mean PET in spring in Poland in the years 1966–2020 (for decades)

Station	1966–1970	1971–1980	1981–1990	1991–2000	2001–2010	2011–2020	Station	1966–1970	1971–1980	1981–1990	1991–2000	2001–2010	2011–2020
Białystok	9.7	7.6	10.3	9.9	10.1	10.9	Opole	11.7	11.4	13.4	11.8	12.3	13.2
Bielsko-Biała	9.7	9.3	10.5	9.6	11.4	11.7	Poznań	8.3	8.6	10.0	10.6	10.7	11.3
Chojnice	7.0	7.6	9.0	8.5	9.0	9.6	Racibórz	13.1	12.3	11.8	11.5	11.4	12.0
Gorzów Wielkopolski	9.2	9.1	10.3	11.1	11.8	12.5	Rzeszów	9.4	9.8	10.8	10.9	11.2	11.6
Hel	6.0	6.1	7.6	7.5	7.7	8.4	Siedlce	9.3	9.1	9.5	9.5	10.2	11.2
Jelenia Góra	9.6	9.5	10.8	11.5	11.0	11.2	Słubice	9.7	10.6	11.5	12.7	12.5	12.3
Kalisz	10.2	9.7	10.3	10.1	10.4	11.2	Sulejów	8.8	9.0	10.0	10.0	10.5	11.2
Katowice	9.8	10.1	10.9	11.4	11.9	12.6	Suwałki	6.2	5.6	7.3	8.2	8.1	9.0
Kielce	10.3	9.2	10.1	10.1	10.7	11.2	Szczecin	8.0	8.8	10.1	10.3	10.3	11.4
Kłodzko	10.2	10.2	10.3	10.7	10.5	11.0	Świnoujście	5.9	5.7	6.8	8.3	8.9	9.1
Kołobrzeg	5.4	6.0	7.7	7.8	8.4	8.5	Terespol	8.5	9.0	10.9	10.3	10.9	11.7
Koszalin	6.0	7.0	7.7	9.0	8.7	9.3	Toruń	9.1	9.3	10.8	10.7	11.1	12.3
Kraków	10.2	10.4	11.3	11.4	11.8	11.9	Ustka	4.4	5.3	6.6	6.1	5.3	5.8
Lesko	9.1	9.6	9.3	9.3	10.6	11.2	Warszawa	8.5	8.6	9.7	9.6	10.3	11.6
Lublin	9.4	8.7	9.9	9.6	10.4	11.0	Włodawa	9.3	8.9	9.3	9.4	10.0	10.7
Łeba	4.9	4.4	5.1	5.7	6.0	6.6	Wrocław	10.0	10.5	11.4	11.4	11.6	12.7
Łódź	7.8	8.2	9.8	10.0	10.1	10.9	Zakopane	8.9	9.2	9.7	9.4	9.7	10.0
Mława	7.5	7.0	8.6	8.8	9.3	10.3	Zielona Góra	9.0	9.1	9.9	10.5	10.9	11.6
Olsztyn	7.5	7.6	9.7	9.4	9.5	10.2							

**Fig. 6:**
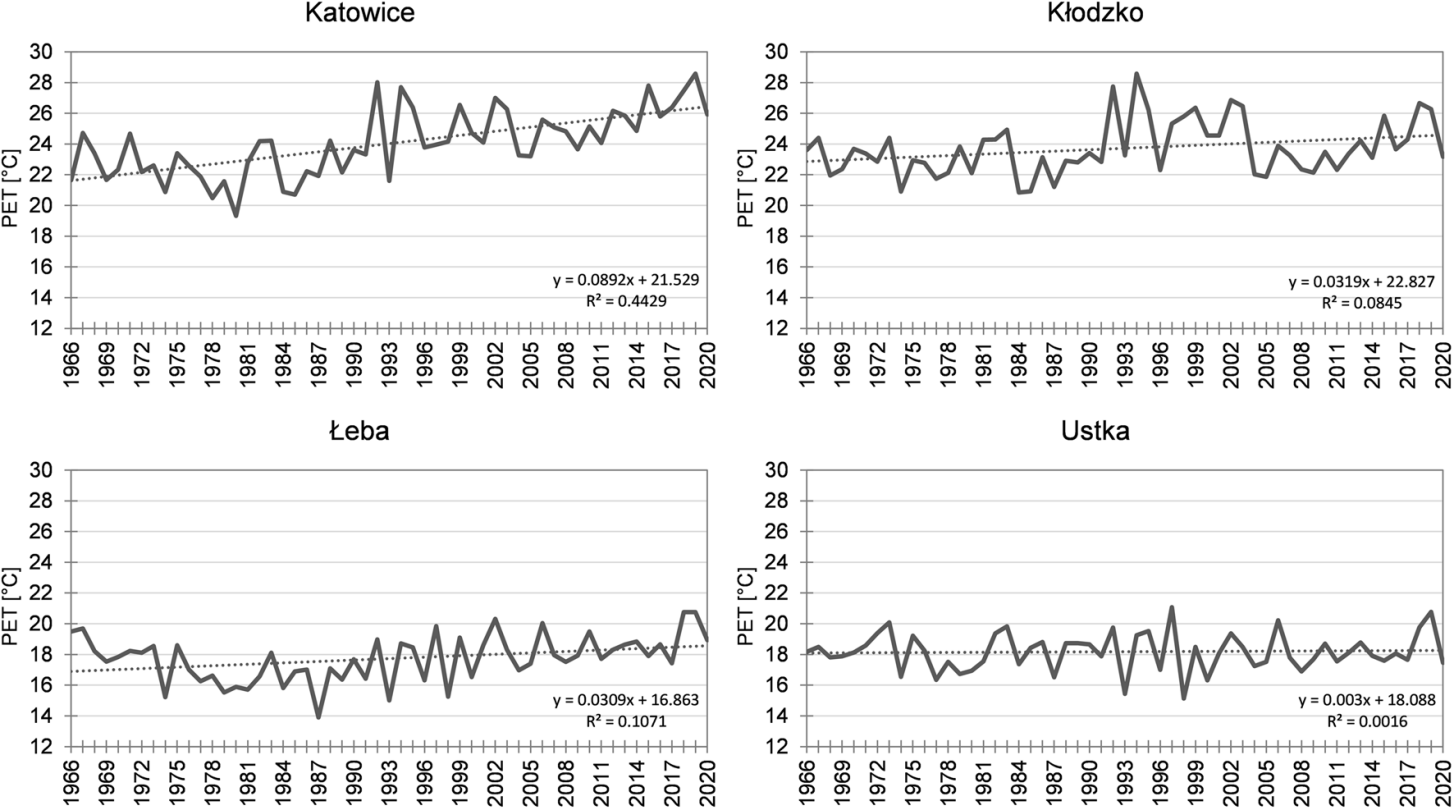
Mean PET in summer in the years 1966–2020 (for 12:00 UTC) in Katowice, Kłodzko, Łeba, and Ustka

In summer in the years 1966–2020, mean PET value was 22.7 °C. The lowest index values were recorded in the northern regions (in Łeba 17.7 °C) of the study area, and the highest in the southern ones, and particularly in the south-western ones (in Opole 25.4 °C) (Fig. 4). The lowest mean PET value for the entire area was recorded in 1980, reaching 19.2 °C. In particular stations, it ranged from 15.9 °C in Łeba to 22.1 °C in Kłodzko (Fig. 6). It was the year with the highest PET value in the analysed multiannual period in 43% of stations. Approximate bioclimatic conditions occurred in 1974, 1978, and 1980. The highest PET value for the study area was recorded in 2019, reaching 26.4 °C, and varied from 20.8 °C in Ustka and Łeba to 30.0 °C in Opole. In 43% of stations in the analysed year, the highest mean seasonal PET value throughout the analysed period was recorded. Approximate conditions were observed in 1992, 2018, 2015, and 2002. In the analysed multiannual period, PET increased in summer throughout the area, with the most intensive course in south-east Poland. In particular stations, the changes varied from 0.03 °C/10 years in Ustka to 0.89 °C/10 years in Katowice (Fig. 6). The recorded changes were statistically significant in 89% of stations. In 57% of stations, PET changes in summer were the most considerable among all seasons. The discussed changes were also evident in mean decade index values (Table 4). In 92% of stations, the highest mean PET were recorded in the period 2010–2020 (in three stations, the same value was recorded in earlier decades). The lowest values were determined in the first or second decade of the analysed period.

**Table 4:** Mean PET in summer in Poland in the years 1966–2020 (for decades)

Station	1966–1970	1971–1980	1981–1990	1991–2000	2001–2010	2011–2020	Station	1966–1970	1971–1980	1981–1990	1991–2000	2001–2010	2011–2020
Białystok	24.5	21.2	23.4	23.8	24.1	24.8	Opole	25.2	23.6	24.7	25.3	26.0	27.5
Bielsko-Biała	22.3	21.2	22.3	22.9	24.7	25.5	Poznań	22.3	21.3	21.8	23.4	23.8	24.3
Chojnice	21.9	20.9	20.7	21.2	21.7	22.3	Racibórz	26.2	24.4	23.6	24.8	24.8	25.7
Gorzów Wielkopolski	23.3	22.2	21.9	23.8	24.6	25.1	Rzeszów	22.9	22.3	23.3	25.0	24.8	26.0
Hel	20.2	19.7	20.0	20.6	20.9	21.4	Siedlce	23.3	22.1	21.8	23.2	24.0	25.5
Jelenia Góra	21.7	21.2	22.6	24.2	23.5	24.2	Słubice	24.1	23.6	23.3	25.1	25.1	25.4
Kalisz	24.3	22.9	22.2	23.1	23.6	24.9	Sulejów	22.3	21.6	21.8	23.1	23.9	25.2
Katowice	22.8	22.0	22.7	25.0	24.8	26.3	Suwałki	21.0	19.3	20.0	22.4	22.1	22.7
Kielce	23.4	22.0	22.2	23.9	24.1	25.5	Szczecin	21.7	21.9	21.9	23.0	22.8	23.6
Kłodzko	23.2	22.7	22.9	25.3	23.7	24.3	Świnoujście	19.3	18.7	18.6	20.7	21.9	21.6
Kołobrzeg	18.6	18.9	19.9	20.4	21.5	21.1	Terespol	22.6	22.3	23.4	24.2	24.8	26.1
Koszalin	19.3	19.2	18.6	20.6	21.1	21.1	Toruń	23.6	23.3	22.6	23.9	24.5	25.8
Kraków	22.8	22.4	23.2	24.6	24.9	25.4	Ustka	18.1	18.0	18.4	18.0	18.2	18.4
Lesko	22.0	21.5	21.1	22.6	23.6	25.1	Warszawa	22.1	21.3	21.5	22.7	23.8	25.4
Lublin	23.2	21.7	22.4	23.6	24.6	25.3	Włodawa	23.8	22.3	21.9	23.5	23.9	24.9
Łeba	18.6	17.0	16.5	17.5	18.5	18.8	Wrocław	23.5	23.3	23.5	24.3	24.8	26.3
Łódź	21.9	21.2	21.7	23.1	23.5	24.5	Zakopane	21.0	21.6	21.5	23.4	22.8	23.7
Mława	21.8	20.1	20.5	22.2	22.8	23.9	Zielona Góra	23.1	21.8	21.7	23.0	23.6	24.6
Olsztyn	22.5	21.3	21.9	22.6	22.8	23.5							

**Fig. 7:**
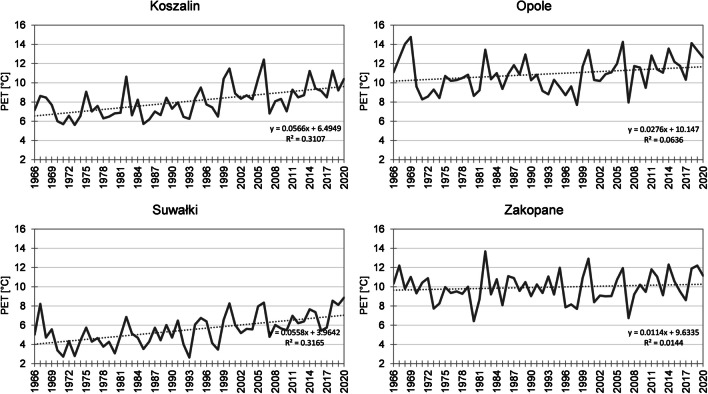
Mean PET in autumn in the years 1966–2020 (for 12:00 UTC) in Koszalin, Opole, Suwałki, and Zakopane

In autumn in the years 1966–2020, mean PET value was 8.7 °C. Although it was lower by 1.0 °C than in spring, the spatial distribution of the isolines was approximate to the spring conditions (Fig. 4). An evident difference was observed in the case of the Baltic Sea coast, where autumn values were higher, which is typical of marine climate. PET values in the study area increased from the north-east (5.5 °C in Suwałki) towards the south-west (10.9 °C in Opole) (Fig. 7). More than 10.0 °C was also recorded in Raciborz, Jelenia Góra, Słubice, Kraków, and Wrocław. Based on mean PET calculated for the entire area, the season with minimum PET was autumn 1998 (mean 6.0 °C). The value in particular stations varied from 3.5 °C in Suwałki to 8.2 °C in Jelenia Góra. Considering the year when the lowest mean PET value throughout the multiannual period was recorded in the highest number of stations, year 1993 deserves attention (mean 6.3 °C). The mean index value varied from 2.6 °C in Suwałki to 11.1 °C in Zakopane. In 1993, in 43% of stations (primarily in north and north-east Poland), the lowest PET value in the multiannual period was recorded, and in 1998 in 30% of stations (primarily in central and south Poland). Except for the station in Raciborz, PET increased in the study area in autumn in the analysed period. The increase progressed the fastest in the north-western and north-eastern regions of the country. In particular stations, the increase varied from 0.03 °C/10 years in Kłodzko to 0.57 °C/10 years in Koszalin (Fig. 7). In 84% of the stations, the changes were statistically significant. In 70% of stations, changes in PET in autumn were the lowest among all seasons. In all stations, the highest mean PET was recorded in the last decade, and the lowest in 95% of stations in the first decade (in two stations, the same value was recorded in subsequent decades) (Table 5).

**Table 5:** Mean PET in autumn in Poland in the years 1966–2020 (for decades)

Station	1966–1970	1971–1980	1981–1990	1991–2000	2001–2010	2011–2020	Station	1966–1970	1971–1980	1981–1990	1991–2000	2001–2010	2011–2020
Białystok	8.4	5.8	7.6	6.8	7.9	8.9	Opole	12.4	9.6	11.0	10.0	11.0	12.3
Bielsko-Biała	10.7	8.3	9.5	8.7	10.6	11.5	Poznań	9.0	7.4	8.4	8.2	8.9	10.1
Chojnice	7.1	6.0	6.8	6.2	7.0	8.1	Racibórz	13.6	10.4	10.2	9.8	10.3	11.6
Gorzów Wielkopolski	9.9	7.9	8.6	8.5	9.9	10.7	Rzeszów	9.7	7.9	9.1	8.9	9.3	10.4
Hel	7.9	6.8	7.5	6.9	8.0	8.8	Siedlce	8.9	6.8	7.2	6.9	8.4	9.6
Jelenia Góra	10.3	9.6	11.3	10.9	10.4	11.5	Słubice	11.0	9.8	10.3	10.5	10.9	11.1
Kalisz	10.8	8.1	8.6	7.9	8.8	10.0	Sulejów	9.3	7.2	8.2	7.9	9.1	10.3
Katowice	10.3	8.2	9.4	9.6	10.3	11.6	Suwałki	5.4	4.0	5.0	5.5	6.1	7.1
Kielce	10.5	7.6	8.3	8.2	9.2	10.4	Szczecin	8.6	7.9	8.7	8.4	9.0	10.2
Kłodzko	10.8	8.9	9.2	9.4	9.3	10.0	Świnoujście	8.4	7.3	7.7	8.1	9.2	10.0
Kołobrzeg	8.3	7.5	8.7	8.5	9.6	10.0	Terespol	8.1	7.0	8.2	7.6	9.0	9.8
Koszalin	7.6	6.8	7.4	8.2	8.7	9.6	Toruń	9.4	7.8	8.3	8.0	9.4	10.4
Kraków	10.7	9.0	10.4	10.3	10.7	11.2	Ustka	8.2	6.9	7.8	7.2	7.8	8.5
Lesko	10.5	8.1	8.4	8.3	9.4	11.1	Warszawa	8.5	6.7	7.4	6.8	8.6	10.0
Lublin	9.4	6.8	7.6	7.2	8.8	9.6	Włodawa	9.0	6.9	7.0	6.8	8.3	9.1
Łeba	8.1	6.3	6.3	6.6	7.7	8.3	Wrocław	10.5	9.5	10.3	9.5	10.2	11.9
Łódź	8.3	6.7	7.8	7.7	8.7	9.8	Zakopane	10.5	9.2	10.2	9.9	9.4	10.8
Mława	7.3	5.3	6.4	6.2	7.3	8.5	Zielona Góra	9.1	7.9	8.3	8.1	9.0	10.3
Olsztyn	7.7	6.3	7.6	7.0	7.6	8.7							

**Fig. 8:**
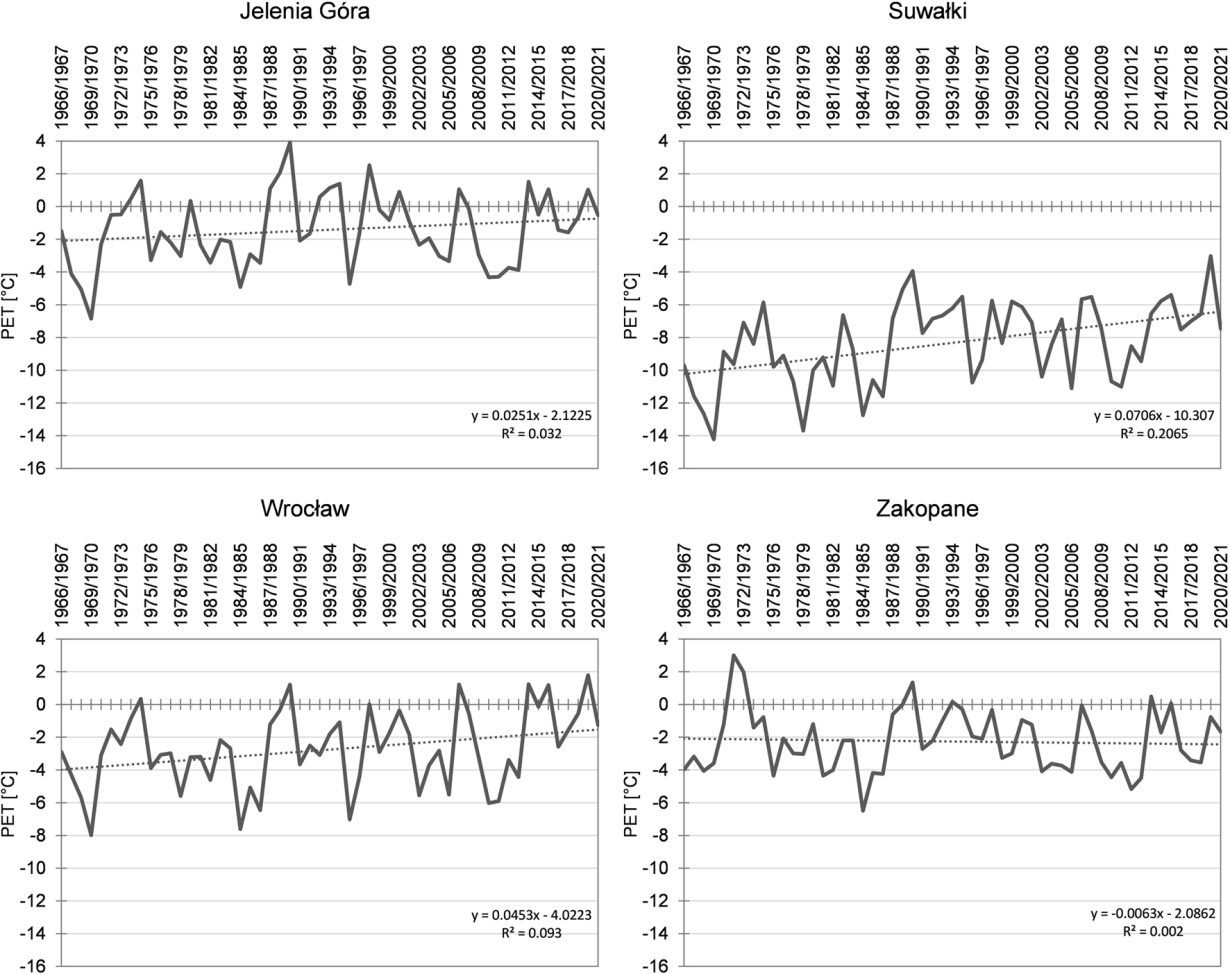
Mean PET in winter in the years 1966–2020 (for 12:00 UTC) in Jelenia Góra, Suwałki, Wrocław, and Zakopane

In winter in the years 1966/1967–2020/2021, mean PET value was − 4.4 °C. In that season, the course of PET isolines was different than in the case of earlier seasons. Over a considerable area, they showed an approximately longitudinal course (Fig. 4). The lowest index values were recorded in north-eastern Poland (− 8.5 °C in Suwałki) and the highest in the south-western and western regions of the country (− 1.4 °C in Jelenia Góra) (Fig. 8). The lowest mean PET value was recorded in winter 1969/1970. It reached − 9.0 °C. In particular stations, it ranged from − 14.2 °C in Suwałki to − 3.5 °C in Zakopane. Below − 10.0 °C was also recorded in Mława, Olsztyn, Warsaw, Terespol, Chojnice, Łódź, Toruń, and Włodawa. In 57% of stations, it was a season with the lowest mean PET in the entire multiannual period. Equally unfavourable bioclimatic conditions were recorded in winter 1984/1985, when the lowest mean PET was recorded in 24% of stations and the mean index value for the entire area reached − 8.7 °C. The highest mean PET value was recorded in winter 2019/2020, reaching − 0.4 °C. In particular stations, mean seasonal value of the index ranged from − 3.0 °C in Suwałki to 1.8 °C in Wrocław. In 68% of stations, it was a season with the highest mean PET in the analysed period. With the exception of two stations (Racibórz, Zakopane), an increase in PET was recorded in winter. It was the most intensive in north-east and north-west Poland. In particular stations, the increase ranged from 0.05 °C/10 years in Kłodzko to 0.71 °C/10 years in Suwałki (Fig. 8). The recorded changes were statistically significant in 73% of stations. In 8% of stations, changes recorded in winter were the greatest among all seasons, and the lowest in 22% of stations. In 84% of stations, the highest mean index value was recorded in the last decade (Table 6). Unlike in the case of earlier seasons of the year, no single dominant decade was identified. The values were recorded in decades: 1971–1980 or 1981–1990 or 2001–2010 (in five stations, the lowest value was observed in two decades). The lowest value was not recorded only in two decades, namely 1991–2000 and 2010–2020.

**Table 6:** Mean PET in winter in Poland in the years 1966/1967–2020/2021 (for decades)

Station	1966/1967–1970/1971	1971/1972–1980/1981	1981/1982–1990/1991	1991/1992–2000/2001	2001/2002–2010/2011	2011/2012–2020/2021	Station	1966/1967–1970/1971	1971/1972–1980/1981	1981/1982–1990/1991	1991/1992–2000/2001	2001/2002–2010/2011	2011/2012–2020/2021
Białystok	− 8.6	− 7.5	− 6.4	− 5.7	− 7.0	− 5.2	Opole	− 3.8	− 2.4	− 2.2	− 2.4	− 3.2	− 1.3
Bielsko-Biała	− 4.7	− 2.7	− 3.2	− 3.1	− 3.4	− 2.1	Poznań	− 6.2	− 4.7	− 4.4	− 3.6	− 4.9	− 2.9
Chojnice	− 7.8	− 6.3	− 6.1	− 5.4	− 6.4	− 4.7	Racibórz	− 1.9	− 1.6	− 3.4	− 2.9	− 4.1	− 2.3
Gorzów Wielkopolski	− 5.0	− 4.4	− 4.4	− 3.2	− 3.8	− 2.0	Rzeszów	− 6.8	− 5.4	− 5.1	− 4.4	− 5.5	− 3.8
Hel	− 6.9	− 5.1	− 4.8	− 4.2	− 4.8	− 3.5	Siedlce	− 7.6	− 6.1	− 6.5	− 5.6	− 6.4	− 4.6
Jelenia Góra	− 4.0	− 1.1	− 1.4	− 0.2	− 2.2	− 0.9	Słubice	− 4.1	− 2.4	− 2.6	− 1.3	− 2.5	− 1.5
Kalisz	− 4.9	− 4.4	− 4.7	− 4.3	− 5.3	− 3.4	Sulejów	− 6.7	− 5.3	− 5.4	− 4.6	− 5.4	− 3.6
Katowice	− 5.2	− 3.6	− 4.1	− 3.2	− 3.8	− 2.0	Suwałki	− 11.4	− 9.3	− 8.5	− 7.1	− 8.4	− 6.7
Kielce	− 5.7	− 4.5	− 5.1	− 4.4	− 5.1	− 3.3	Szczecin	− 5.6	− 4.2	− 3.8	− 3.1	− 3.9	− 1.9
Kłodzko	− 4.3	− 2.4	− 3.8	− 3.0	− 3.8	− 2.8	Świnoujście	− 5.5	− 4.2	− 4.5	− 3.2	− 3.6	− 1.9
Kołobrzeg	− 5.9	− 4.0	− 3.7	− 3.1	− 3.3	− 2.2	Terespol	− 8.7	− 6.4	− 5.7	− 5.3	− 6.2	− 4.5
Koszalin	− 6.5	− 4.6	− 4.7	− 3.2	− 4.3	− 2.7	Toruń	− 6.6	− 5.0	− 4.8	− 4.0	− 4.7	− 2.9
Kraków	− 5.8	− 3.3	− 3.4	− 2.6	− 4.1	− 2.5	Ustka	− 6.1	− 4.6	− 4.3	− 4.0	− 4.7	− 3.4
Lesko	− 4.8	− 2.7	− 4.6	− 4.0	− 4.3	− 3.1	Warszawa	− 8.1	− 6.1	− 5.9	− 5.3	− 5.8	− 3.5
Lublin	− 7.4	− 6.5	− 6.6	− 5.9	− 6.4	− 4.7	Włodawa	− 8.1	− 6.6	− 6.8	− 6.0	− 6.8	− 5.1
Łeba	− 6.2	− 5.2	− 5.6	− 4.5	− 4.8	− 3.8	Wrocław	− 4.8	− 2.6	− 3.3	− 2.5	− 3.4	− 1.0
Łódź	− 7.5	− 5.8	− 5.7	− 4.9	− 5.6	− 3.5	Zakopane	− 3.2	− 1.5	− 2.5	− 1.5	− 3.0	− 2.3
Mława	− 9.2	− 7.4	− 7.1	− 6.3	− 7.2	− 5.3	Zielona Góra	− 5.7	− 4.1	− 4.7	− 3.7	− 4.5	− 2.7
Olsztyn	− 8.3	− 6.4	− 5.9	− 5.1	− 6.5	− 4.7							

**Fig. 9:**
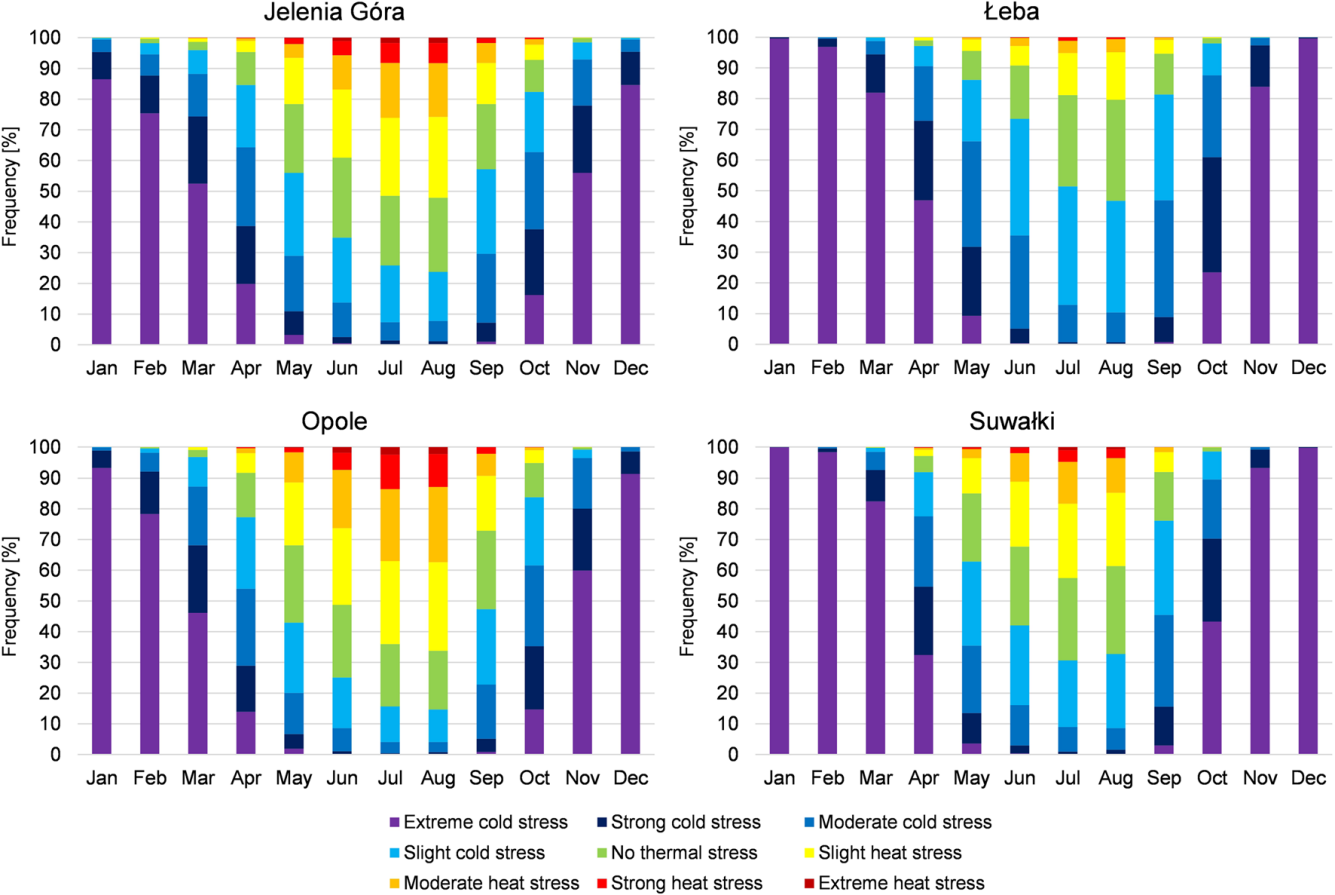
Share of days with particular PET categories during the year in selected stations in Poland in the years 1966–2020 (for 12:00 UTC)

In spring in the analysed years, cold stress categories were dominant. Their share changed in particular months of the season. In March, those categories constituted from 96.0% of days in Jelenia Góra to 99.9% of days in Ustka and Łeba (Fig. 9). Among these days, days with extreme cold stress were recorded, and their share varied from 46.2% of all days in Opole to 82.5% of all days in Suwałki. In 19% of stations in the analysed period, no days from heat stress categories were recorded. In April, the share of cold stress categories decreased in favour of days with no thermal stress and days with heat stress. Cold stress categories constituted from 77.3% of days in Opole to 97.2% of days in Łeba. Days with no thermal stress constituted from 1.8% of days in Łeba to 14.4% of days in Opole. The lowest share of such days was observed at the coast, and the greatest in the south and west of Poland. Days with heat stress were recorded in all stations. Their share varied from 1.0% of days in Łeba and Hel to 8.3% of days in Opole. In the last of the spring months, in 22% of stations, cold stress categories constituted less than half of all days. In particular stations, their share varied from 42.9% of days in Opole to 88.2% of days in Ustka. Days with slight cold stress and moderate cold stress occurred the most frequently. A similar frequency was determined for the occurrence of days with no thermal stress and days with heat stress. The share of the former ranged from 7.1% of days in Ustka to 27.3% of days in Białystok and Włodawa. In the latter group, the share varied from 4.5% of days in Łeba to 31.8% of days in Opole. In all the analysed stations, days with strong heat stress were recorded, and in 62% of stations days with extreme heat stress (their highest share was observed in Raciborz).

In summer, heat stress or no thermal stress categories were dominant. In the first summer month, only in 13% of stations, days with cold stress constituted more than half of all days. In particular stations, their share varied from 25.1% of days in Opole to 73.4% of days in Łeba (Fig. 9). An approximate share of such days was also recorded in Ustka. Among days with cold stress, days with slight cold stress were generally dominant. In 73% of stations, days with heat stress occurred the most frequently. The share of days in the said category varied from 8.8% in Ustka to 51.3% in Opole (and only in this station they constituted more than half of all days). Except for the coast, the share of days of that category was uniform throughout the study area, with an increased share in the south. No days with extreme heat stress were only recorded in 8% of stations, and the highest number of such days was determined in Opole. Days with no thermal stress constituted from 17.4% of days in Łeba to 31.8% of days in Szczecin. In July, days with heat stress evidently dominated. In 73% of stations, they accounted for more than half of all days. In particular stations, the share of days of the analysed category ranged from 18.8% in Łeba to 64.0% in Opole. Days with slight and moderate heat stress occurred the most frequently. No days with extreme heat stress were recorded only in 5% of stations. Such days were observed the most frequently in Opole and Kraków. Days with no thermal stress constituted from 20.3% of days in Opole to 36.1% of days in Świnoujście. Days with cold stress constituted from 15.7% of days in Opole to 51.4% of days in Łeba (only in this station, days of this category accounted for more than half of all days). In this category of thermal stress, days with slight cold stress were the most frequent. Days with extreme cold stress were recorded in 16% of stations. In August, the share of particular categories of thermal stress was approximate to that observed in July. The share of days with heat stress was dominant, and in 68% of stations, such days accounted for more than half of all days. In particular stations, their share varied from 20.4% of days in Łeba to 66.2% of days in Opole. Days with slight and moderate heat stress occurred the most frequently. In all stations, days with all categories of heat stress were recorded, including extreme heat stress, the most frequently observed in Opole. Days with no thermal stress constituted from 19.1% of days in Opole to 36.1% of days in Kołobrzeg. Days with cold stress constituted from 14.7% of days in Opole to 46.7% of days in Łeba. In this category of thermal stress, days with slight cold stress were the most numerous. Days with extreme cold stress were observed in 35% of stations.

In autumn, cold stress categories were dominant again. They were recorded the most frequently at the coast, and the most seldom in the southern regions of the country. The smallest share of days with cold stress was determined for September. In particular stations, the share of such days varied from 47.3% in Opole to 81.3% in Łeba (Fig. 9). Only in Opole such days did not exceed half of all days. Days with slight and moderate cold stress were recorded the most frequently. Days with extreme cold stress occurred in all stations and were the most frequent in Zakopane. Days with no thermal stress constituted from 13.3% of days in Łeba to 25.5% of days in Opole. The share of days with heat stress ranged from 5.4% in Łeba to 27.2% in Opole. In the scope of the said category, days with slight heat stress occurred the most frequently. Days with extreme heat stress were recorded in 46% of stations. In October, the share of days with cold stress varied from 80.9% in Kraków to 98.8% in Hel. Days with moderate and strong cold stress were recorded the most frequently. Days with extreme cold stress were equally frequent, reaching from 12.1% of days in Słubice to 43.3% of days in Suwałki. Days with no thermal stress constituted from 1.0% of days in Hel to 12.0% of days in Kraków. Days with heat stress were even more rare, constituting from 0.1% of days in Suwałki to 7.2% of days in Jelenia Góra. In November, in 27% of stations, all days were classified as days with cold stress, and the lowest number of such days was observed in Kraków, reaching 98.5% of days. Days with extreme cold stress were recorded the most frequently, from 56.0% of all days in Jelenia Góra to 93.3% of all days in Suwałki. In 30% of stations, no days with no thermal stress were recorded, and in 62% of stations, no days with heat stress.

In all months of the winter season, almost all days were characterised by cold stress. One hundred percent of days with cold stress was not recorded in December only in Opole, and in January only in Opole and Lesko. In the aforementioned stations, 0.1% of days were classified as days with no thermal stress. The conditions were somewhat different in February. The share of days with cold stress ranged from 98.2% of days to 100% days. In 57% of stations, days with no thermal stress were recorded, and in 13% of stations, days with heat stress. Among days with cold stress, days with extreme cold stress occurred the most frequently in each month. In December, their share varied from 84.6% of days to 99.8% of days, in January from 86.5% of days to 99.9% of days, and in February from 75.4% of days to 98.4% of days. In each of the said months, the minimum share was observed in Jelenia Góra, and maximum in Suwałki (Fig. 9).

The study revealed considerable changes in the occurrence of conditions causing heat and cold stress, but also conditions described as no thermal stress. In 95% of stations, a decrease in the frequency of occurrence of conditions causing cold stress was determined, and in 89% of stations, the recorded changes were statistically significant. Over a major area, the decreases were at an approximate level (4–6 days/10 years), and the greatest decreases were observed in Świnoujście (7.5 days/10 years) and Katowice (7.1 days/10 years). In 89% of stations, the frequency of occurrence of days with no thermal stress increased, and in 92% of stations, an increase in the frequency of occurrence of conditions causing heat stress was recorded. In the case of the former category of days, the occurring changes were statistically significant in 57% of stations, and in the latter case in 70% of stations. The greatest increase in the frequency of occurrence of days with heat stress was observed in Terespol (5.6 days/10 years) and Katowice and Lesko (5.0 days/10 years).

## Summary and discussion

On the basis of the conducted research, it revealed high variability of bioclimatic conditions in Poland both in spatial and in temporal terms. The lowest mean annual PET values were recorded in the north and north-east of the country, and the highest in the south-west of Poland. The obtained results are in line with earlier studies on bioclimatic conditions in Poland based on other indices, e.g. UTCI (Kuchcik et al. 2021; Tomczyk and Bednorz 2023). Mean annual distribution of PET values was approximate to the distribution of mean annual air temperature in Poland (Kejna and Rudzki 2021; Ustrnul et al. 2021; Tomczyk 2022). Low PET values in coastal stations result from the cooling effect of the Baltic Sea and increased wind speed and air humidity (Kejna and Rudzki 2021; Wibig 2021, 2022; Wypych 2022). In north-east Poland, the main factor affecting the index value were low air temperature values—next to the mountains, the area is among the coldest areas in the country (Kejna and Rudzki 2021; Ustrnul et al. 2021; Tomczyk 2022). On the background of the analysed multiannual period, 2 years particularly stood out, namely 1980 and 2018, with recorded lowest and highest PET values, respectively. In those years, the lowest and highest mean annual UTCI values were also recorded over a major part of Poland (Tomczyk and Bednorz 2023). The said years were among the coldest and warmest years in the recent decades in Poland (Tomczyk and Bednorz 2020; Matuszko et al. 2022). The conducted research showed an increase in mean annual PET values, and the most intensive changes were recorded in the north-western and north-eastern parts of the country. The obtained results are in line with earlier studies on bioclimatic conditions in Poland, and the differences in the rate of changes result from the adopted multiannual periods and indices (Tomczyk and Bednorz 2020; Matuszko et al. 2022). Changes in mean annual UTCI in the period 1951–2018 over a predominant area of Poland were statistically significant, and an increase in the index value was determined in each of the analysed stations (Kuchcik et al. 2021). The highest increase occurred in Suwałki (0.90 °C/10 years), Tarnów (0.89 °C/10 years), and Świnoujście (0.80 °C/10 years). According to Marosz et al. (2023), 9 warmest years in Poland since 1951 occurred after 2000. The above changes caused in changes in bioclimatic conditions. In a large majority of stations, the highest mean annual PET value in the studied multiannual period was recorded in the twenty-first century.

The spatial distribution of mean annual PET values was approximate to the spatial distribution of mean values for particular seasons. The highest values were recorded in the south-western regions of the country. The situation was somewhat different in the case of the lowest values. In spring and summer, they were recorded in the northern regions—at the coast of the Baltic Sea, and in autumn and winter in the north-eastern regions. Such a distribution of PET values was a consequence of the cooling effect of the sea in spring and summer, and its warming effect in autumn and winter in the coastal zone. A similar spatial distribution of the lowest and highest PET values was also observed during heat waves in Poland (Tomczyk et al. 2020). Except for two stations, an increase in PET values was recorded in all seasons of the year in the analysed multiannual period. The changes showed no uniform course in a year. In 57% of stations, the most intensive changes were recorded in summer, and in 70% of stations, the smallest changes were observed in autumn. The level of changes in the index values is evident in the comparison of the obtained results with results of research by Błażejczyk and Matzarakis (2007) covering the period 1961–1990. In all seasons of the year, the highest values were recorded in this study, and the differences are at a level of several degrees Celsius.

The study revealed changes in the frequency of occurrence of days with cold and heat stress, as well as days with no thermal stress. The most intensive changes were determined for days with cold stress. A decrease in the number of days in this category translated into an increase in the number of days with no thermal stress and days with heat stress. Changes in the frequency of occurrence of days characteristic in biometeorological terms were addressed in earlier studies regarding summer (Tomczyk and Owczarek 2020; Krzyżewska et al. 2021a, b; Miszuk 2021; Tomczyk et al. 2023) and winter (Wereski et al. 2020; Miszuk 2021; Owczarek and Tomczyk 2022; Tomczyk et al. 2023). The authors of the cited studies directly pointed to an increase in the number of days with heat stress and a decrease in the number of days with cold stress. According to Kuchcik (2021) based on UTCI, an increase in heat stress contributed to an increase in death risk. The author evidenced that the > 38 °C risk of death increased by 25–30% in central Poland.

According to Tomczyk et al. (2022) and Piniewski et al. (2017), the maximum daily air temperature and the number of hot days will increase by the end of the twenty-first century. Due to this, a further increase in conditions causing heat stress should be expected, and implication will be in lead in the increase of morbidity and mortality. This is also confirmed by research by Błażejczyk et al. (2013) that predicted an increase in the number of days with heat stress (according to UTCI) in Warsaw at a rate of 0.9 days/10 years in the period 2000–2100. Similar changes have been found in other regions of Europe, e.g. in south-western Germany (Matzarakis and Endler 2010). In the following decades of the twenty-first century, there will be more and more days with strong heat stress and extreme heat stress. During this time, there will be less and less days with strong cold stress and extreme cold stress.

## Conclusions

The conducted research showed that the progressing climate changes also translated into a change in bioclimatic conditions in Poland. An increase in PET values was determined both in the annual scale and in particular seasons of the year. During the year, statistically significant changes over the largest area were recorded in summer, and the smallest in winter. The aforementioned changes translated into an increase in the number of days with no thermal stress and days with heat stress, as well as a decrease in the number of days with cold stress. This suggests that in the cool seasons, the strenuous character of bioclimatic conditions resulting from cold stress will decrease, and an increase in the strenuous character of such conditions should be expected in summer due to increasingly frequent occurrence of heat stress. Unfortunately, a limitation of the study is the lack of determination of the effect of the recorded conditions on human health and life. This results from high level of inaccessibility of medical data. In the opinion of the authors, the development of research in that direction should be treated as a priority in the future.

## Data Availability

The obtained data can be made available on request of interested parties under the condition of approval of the request by the authors of the article.

## References

[CR1] Bal S, Matzarakis A (2022). Temporal analysis of thermal bioclimate conditions between Kolkata (India) and its three neighbouring suburban sites. Theor Appl Climatol.

[CR2] Basarin B, Lukić T, Mesaroš M, Pavić D, Đorđević J, Matzarakis A (2018). Spatial and temporal analysis of extreme bioclimate conditions in Vojvodina. Northern Serbia Int J Climatol.

[CR3] Błażejczyk K (1994). New climatological- and -physiological model of the human heat balance outdoor (MENEX) and its applications in bioclimatological studies in different scales. Zeszyty IGiPZ PAN.

[CR4] Błażejczyk K (2004) Bioklimatyczne uwarunkowania rekreacji i turystyki w Polsce. Prace Geograficzne, Instytut Geografii i Przestrzennego Zagospodarowania (IGiPZ) PAN, 192, 291.

[CR5] Błażejczyk K (2005) New indices to assess thermal risks outdoors. In: Holmér I, Kuklane K, Gao Ch (ed), Environmental ergonomics XI, Proc. Of the 11th International Conference, 22–26 May, 2005 Ystat, Sweden, 222–225.

[CR6] Błażejczyk K (2007) Multiannual and seasonal weather fluctuations and tourism in Poland. In: Amelung B, Błażejczyk K, Matzarakis A (ed), Climate change and tourism assessment and copying strategies, Maastricht – Warsaw – Freiburg: 69–90.

[CR7] Błażejczyk K, Matzarakis A (2007). Assessment of bioclimatic differentiation of Poland based on the human heat balance. Geogr Pol.

[CR8] Błażejczyk K, Twardosz R (2023) Secular changes (1826–2021) of human thermal stress according to UTCI in Kraków (southern Poland). Int J Climatol10.1002/joc.8083

[CR9] Błażejczyk K, Broede P, Fiala D, Havenith G, Holmér I, Jendritzky G, Kampmann B, Kunert A (2010). Principles of the new Universal Thermal Climate Index (UTCI) and its application to bioclimatic research in European scale. Miscellanea Geographica.

[CR10] Błażejczyk K, Epstein Y, Jendritzky G, Staiger H, Tinz B (2012). Comparison of UTCI to selected thermal indices. Int J Biometeorol.

[CR11] Błażejczyk K, Idzikowska D, Błażejczyk A (2013). Forecast changes for heat and cold stress in Warsaw in the 21st century, and their possible influence on mortality risk. Pap Glob Chang.

[CR12] Ferrari J, Shiue I, Seyfang L, Matzarakis A, Lang W, Austrian Stroke Registry C (2015). Weather as physiologically equivalent was not associated with ischemic stroke onsets in Vienna, 2004–2010. Environ Sci Pollut Res Int.

[CR13] Fröhlich D, Gangwisch M, Matzarakis A (2019). Effect of radiation and wind on thermal comfort in urban environments - Application of the RayMan and SkyHelios model. Urban Climate.

[CR14] Höppe PR (1999). The physiological equivalent temperature—a universal index for the bioclimatological assessment of the thermal environment. Int J Biometeorol.

[CR15] Houghton FC, Yaglou CP (1923). Determining equal comfort lines. J Am Soc Heat Vent Eng.

[CR16] IPCC, 2021. Climate change 2021: the physical science basis. Contribution of Working Group I to the Sixth Assessment Report of the Intergovernmental Panel on Climate Change. In: Masson-Delmotte V, Zzhai P, Pirani A, Connors SL, Péan C, Berger S, Caud N, Chen YY, Goldfarb L, Gomis MI, Huang M, Leitzell K, Lonnoy E, Matthews JBR, Maycock TK, Waterfield T, Yyelekçi O, Zhou B (ed). Cambridge University Press.

[CR17] Kejna M, Rudzki M (2021). Spatial diversity of air temperature changes in Poland in 1961–2018. Theor Appl Climatol.

[CR18] Kolendowicz L, Półrolniczak M, Szyga-Pluta K, Bednorz E (2018). Human-biometeorological conditions in the southern Baltic coast based on the universal thermal climate index (UTCI). Theor Appl Climatol.

[CR19] Krzyżewska A, Wereski S, Demczuk P (2019) Biometeorological conditions during an extreme heatwave event in Poland in August 2015. Weather10.1002/wea.3497

[CR20] Krzyżewska A, Wereski S, Dobek M (2021). Summer UTCI variability in Poland in twenty-first century. Int J Biometeorol.

[CR21] Krzyżewska A, Wereski S, Dobek M (2021). Summer UTCI variability in Poland in the twenty-first century. Int J Biometeorol.

[CR22] Kuchcik M (2021). Mortality and thermal environment (UTCI) in Poland—long-term, multi-city study. Int J Biometeorol.

[CR23] Kuchcik M, Błażejczyk K, Halaś A (2021) Changes in bioclimatic indices. In: Falarz M (ed) Climate change in Poland. Springer Climate, pp 471–491. 10.1007/978-3-030-70328-8_19

[CR24] Mann HB (1945). Nonparametric tests against trend. Econometrica.

[CR25] Marosz M, Miętus M, Biernacik D (2023). Features of multiannual air temperature variability in Poland (1951–2021). Atmosphere.

[CR26] Masterson J, Richardson FA (1979) Humidex, a method of quantifying human discomfort due to excessive heat and humidity, vol 151. Environment Canada, Downsview, Ontario, pp 1–79

[CR27] Matuszko D, Bartoszek K, Soroka J (2022). Relationships between sunshine duration and air temperature in Poland. Geogr Pol.

[CR28] Matzarakis A (2013). Stadtklima vor dem Hintergrund des Klimawandels. Gefahrstoffe - Reinhaltung Der Luft.

[CR29] Matzarakis A, Amelung B (2008) Physiologically equivalent temperature as indicator for impacts of climate change on thermal comfort of humans. In: Thomson MC et al (ed), Seasonal forecasts, climatic change and human health. Advances in global change research 30. Springer, Berlin, 161–172.

[CR30] Matzarakis A, Endler C (2010). Climate change and thermal bioclimate in cities: impacts and options for adaptation in Freiburg. Germany Int J Biometeorol.

[CR31] Matzarakis A, Fröhlich D (2018) Influence of urban green on human thermal bioclimate – application of thermal indices and micro-scale models. Acta Horticul. 10.17660/ActaHortic.2018.1215.1

[CR32] Matzarakis A, Mayer H, Iziomon MG (1999). Applications of a universal thermal index: Physiological Equivalent Temperature. Int J Biometeorol.

[CR33] Matzarakis A, Rutz F, Mayer H (2007). Modelling Radiation fluxes in simple and complex environments – application of the RayMan model. Int J Biometeorol.

[CR34] Matzarakis A, De Rocco M, Najjar G (2009). Thermal bioclimate in Strasbourg—the 2003 heat wave. Theor Appl Climatol.

[CR35] Matzarakis A, Rutz F, Mayer H (2010). Modelling Radiation fluxes in simple and complex environments – basics of the RayMan model. Int J Biometeorol.

[CR36] Matzarakis A, Fröhlich D, Bermon S, Adami PE (2019). Visualization of climate factors for sports events and activities–the Tokyo 2020 Olympic Games. Atmosphere.

[CR37] Matzarakis A, Gangwisch M, Fröhlich D (2021) RayMan and SkyHelios Model. In: Palme M, Salvati A (ed) Urban microclimate modelling for comfort and energy studies. Springer, Cham, 339–361. 10.1007/978-3-030-65421-4_16

[CR38] Mayer H, Höppe P (1987). Thermal comfort of man in different urban environments. Theor Appl Climatol.

[CR39] Milošević D, Dunjić J, Stojsavljević R. et al. (2023) Analysis of long- and short-term biometeorological conditions in the Republic of Serbia. Int J Biometeorol10.1007/s00484-023-02482-8 10.1007/s00484-023-02482-8 37140657

[CR40] Miszuk B (2021). Multi-annual changes in heat stress occurrence and its circulation conditions in the Polish-Saxon border region. Atmosphere.

[CR41] Nastos PT, Matzarakis A (2012). The effect of air temperature and human thermal indices on mortality in Athens. Greece Theor Appl Climatol.

[CR42] Omonijo AG (2017). Assessing seasonal variations in urban thermal comfort and potential health risks using Physiologically Equivalent Temperature: a case of Ibadan, Nigeria. Urban Clim.

[CR43] Owczarek M (2019). The influence of large-scale factors on the heat load on human beings in Poland in the summer months. Theor Appl Climatol.

[CR44] Owczarek M (2021). The influence of air temperature diversity in Central Europe on the occurrence of very strong and extreme cold stress in Poland in winter months. Geogr Pol.

[CR45] Owczarek M, Tomczyk AM (2022). Impact of atmospheric circulation on the occurrence of very strong and extreme cold stress in Poland. Quaest Geogr.

[CR46] Pecelj M, Matzarakis A, Vujadinović M, Radovanović M, Vagić N, Đurić D (2021). Temporal analysis of urban-suburban PET, mPET and UTCI indices in Belgrade (Serbia). Atmosphere.

[CR47] Piniewski M, Mezghani A, Szcześniak M, Kundzewicz Z (2017). Regional projections of temperature and precipitation changes: robustness and uncertainty aspects. Meteorol Zeitschrift.

[CR48] Potchter O, Cohen P, Lin TP, Matzarakis A (2018). Outdoor human thermal perception in various climates: a comprehensive review of approaches, methods and quantification. Sci Total Environ.

[CR49] Potchter O, Cohen P, Lin TP, Matzarakis A (2022). A systematic review advocating a framework and benchmarks for assessing outdoor human thermal perception. Sci Total Environ.

[CR50] Rozbicka K, Rozbicki T (2021). Long-term variability of bioclimatic conditions and tourism potential for Warsaw agglomeration (Poland). Int J Biometeorol.

[CR51] Shevchenko O (2021). Human thermal comfort conditions during heat wave events in Kyiv, Ukraine. Environ Res Eng Manag.

[CR52] Shevchenko O, Snizhko S, Zapototskyi S, Svintsitska H, Matviienko M, Matzarakis A (2022). Long-term analysis of thermal comfort conditions during heat waves in Ukraine. Geogr Pol.

[CR53] Steadman RG (1984). A universal scale of apparent temperature. J Appl Meteorol Climatol.

[CR54] Tomczyk AM, Kolendowicz L, Bednorz E, Tomczyk AM (2019). Fale upałów w Polsce latem 2018 roku. Zmienność klimatu Polski i Europy oraz jej cyrkulacyjne uwarunkowania.

[CR55] Tomczyk AM (2021). Bioclimatic conditions of June 2019 in Poland on a multi-year background (1966–2019). Atmosphere.

[CR56] Tomczyk AM, Tomczyk AM, Bednorz E (2022). Temperatura powietrza. Atlas klimatu Polski.

[CR57] Tomczyk AM, Bednorz E (2020). The extreme year – analysis of thermal conditions in Poland in 2018. Theor Appl Climatol.

[CR58] Tomczyk AM, Bednorz E (2023). Regional and seasonal variability in human thermal stress in Poland. Theor Appl Climatol.

[CR59] Tomczyk AM, Owczarek M (2020). Occurrence of strong and very strong heat stress in Poland and its circulation conditions. Theor Appl Climatol.

[CR60] Tomczyk AM, Bednorz E, Matzarakis A (2020). Human-biometeorological conditions during heatwaves in Poland. Int J Climatol.

[CR61] Tomczyk AM, Piniewski M, Eini MR, Bednorz E (2022). Projections of changes in maximum air temperature and hot days in Poland. Int J Climatol.

[CR62] Tomczyk AM, Bednorz E, Szyga-Pluta K, Owczarek M (2023). Effect of regional baric systems on the occurrence of bioclimatic conditions in Poland. Quaest Geogr.

[CR63] Ustrnul Z, Wypych A, Czekierda D (2021) Air temperature change. In: Falarz M (ed) Climate change in Poland - past, present, future. Springer Climate, pp 275–330. 10.1007/978-3-030-70328-8_11

[CR64] Wereski S, Krzyżewska A, Dobek M (2020). Winter UTCI variability in Poland in the 21st century. Miscellanea Geographica.

[CR65] Wibig J (2021) Change of wind. In: Falarz M (ed) Climate change in Poland - past, present, future. Springer Climate, pp 391–420. 10.1007/978-3-030-70328-8_15

[CR66] Wibig J, Tomczyk AM, Bednorz E (2022). Prędkość wiatru. Atlas klimatu Polski.

[CR67] Wypych A, Tomczyk AM, Bednorz E (2022). Wilgotność powietrza. Atlas klimatu Polski.

